# Modern Aspects of Pernicious Anæmia

**Published:** 1894-02-10

**Authors:** 


### MODERN ASPECTS OF PERNICIOUS ANÆMIA.

THIS disease is a good instance of the advances that have recently been made in clinical medicine, for not only are there many alive who can remember its discovery, but since that took place we have learnt much as to its chemical pathology.

Addison first described it in his work "On Constitutional and Local Effects of Disease of the Suprarenal Capsules," and its discovery is one of the best instances of the genius of that physician, for the disease is so infrequently met with that he must have had a marvellous faculty for observation to have associated the cases with each other. He said, "For a long period I had from time to time met with a very remarkable form of general anæmia, occurring without any discoverable cause whatever—cases in which there had been no previous loss of blood, no chlorosis, no purpura, no renal, splenic, miasmatic, glandular, strumous, or malignant disease. Accordingly, I, in speaking of this form in clinical lecture, and perhaps with little propriety, applied to it the term 'idiopathic' to distinguish it from cases in which there existed some of the usual causes, or concomitants of the anæmic state." Since the time of Addison the disease he here describes has been called pernicious anæmia by most writers.

The next stage in the history of this malady was that many physicians totally misinterpreted the original conception of pernicious anæmia, and applied this term to the most divers conditions. For instance, many German observers stated that women who were profoundly anæmic, either from chlorosis or severe loss of blood, were instances of pernicious anæmia. Thus there was much confusion, for the English observers understood cases of pernicious anæmia when they saw them, and limited the term according to Addison's description, while French and German observers used the term in such a loose way that it meant nothing. Thanks to a very explicit paper by Wilks, and another by Pye-Smith, the profession both abroad and in England were brought to limit the term pernicious anæmia to cases in which no evidence existed "of the usual causes or concomitants of the anæmic state."

Pernicious anæmia is recognised at the bedside by the following signs. Firstly, by there being no evidence of the usual causes of anæmia. Secondly, the colour of the pallor. This is in most cases of a lemon or primrose tint, which is best seen on the face, but in a well-marked case can be observed on any part of the body. It must of course be carefully distinguished from jaundice, but this is readily done by observing the conjunctivæ. Thirdly, the changes in the blood. For a long while it was maintained that the red blood corpuscles underwent peculiar and distinctive changes of shape in this disease. Extended observations have shown that this is an error, and that the changes met with are either due to the mode of preparation of the specimen or may be seen in other diseases. It has also been said that in pernicious anæmia the diminution in the number of the red corpuscles is greater than the diminution of the hæmoglobin, from which it follows that each red blood corpuscle contains more hæmoglobin than is usual. This condition is, however, by no means constant, although it is sometimes observed. Quite commonly the diminution of the hæmoglobin is in excess of the diminution of the number of red corpuscles. There is no characteristic blood state in pernicious anæmia; all that we find is a great loss of red blood corpuscles and hæmoglobin, without any excess of white blood corpuscles. Fourthly, in this disease, as in many of the primary anæmias hæmorrhages are very common, especially in the retina. Fifthly, optic neuritis can be seen in the majority of patients suffering from this malady. Sixthly, pyrexia. Most patients have their temperature raised a little. The usual thing is for it to be about 100 degrees in the morning, and a little above this in the evening. Seventhly, the urine. Much attention has been directed to the colour of this. Addison and the early observers did not describe anything wrong with it. Then it was in reports of cases occasionally noted to be dark, but no special significance was attached to this. Next some described it as being dark in every case. The result has been that for the last three or four years the colour has been very carefully observed, and it has been found that in many cases the urine is especially dark, but that it is quite exceptional to meet with a case in which it is constantly dark. Usually it is dark for a few days or a week, and then is light again for some time, and dark again later on. In a small minority of cases it is never dark. Much dispute has taken place as to the cause of this darkness. All who have paid special attention to the subject admit that the darkness is due to the pigment urobilin. At first it was thought to be due to what is known as pathological urobilin, which is stated to give a characteristic spectrum. This substance can be separated by shaking the urine with chloroform and dissolving in alcohol. An alcoholic solution of chloride of zinc added to the urine gives a green fluorescence, but this test is difficult to obtain. Only one or two observers, however, could convince themselves that this so-called pathological urobilin was present. Others maintained that the dark colour of the urine was due to normal urobilin, and that pathological urobilin did not exist, but that an admixture of other pigmentary bodies to normal urobilin had been mistaken for it. This is very probably true, and we may take it, until it is proved to the contrary, that the dark colour of the urine is due to an excessive amount of urobilin. This is very easily recognised by the broad band it gives between the blue and green parts of the spectrum.

It has been said that there is an excess of iron in the urine in pernicious anæmia, and that the quantity of aromatic sulphates is increased. It has also been stated that some ptomaines which are never met with in the urine in health are found in pernicious anæmia. But these observations about the iron, the aromatic sulphates, and the ptomaines require confirmation.

In our next article we will give an account of the modern views of the pathology, prognosis, and treatment of this disease.

